# Engineering the vasculature of decellularized rat kidney scaffolds using human induced pluripotent stem cell-derived endothelial cells

**DOI:** 10.1038/s41598-019-44393-y

**Published:** 2019-05-29

**Authors:** Osele Ciampi, Barbara Bonandrini, Manuela Derosas, Sara Conti, Paola Rizzo, Valentina Benedetti, Marina Figliuzzi, Andrea Remuzzi, Ariela Benigni, Giuseppe Remuzzi, Susanna Tomasoni

**Affiliations:** 1 Istituto di Ricerche Farmacologiche Mario Negri IRCCS, Centro Anna Maria Astori, Science and Technology Park Kilometro Rosso, 24126 Bergamo, Italy; 2 Department of Chemistry, Materials and Chemical Engineering Giulio Natta, Politecnico di Milano, 20133 Milan, Italy; 3 Department of Industrial Engineering, Bergamo University, 24044 Dalmine, Bergamo Italy; 4 L. Sacco Department of Biomedical and Clinical Sciences, University of Milan, 20122 Milan, Italy

**Keywords:** Kidney, Stem-cell differentiation

## Abstract

Generating new kidneys using tissue engineering technologies is an innovative strategy for overcoming the shortage of donor organs for transplantation. Here we report how to efficiently engineer the kidney vasculature of decellularized rat kidney scaffolds by using human induced pluripotent stem cell (hiPSCs)-derived endothelial cells (hiPSC-ECs). *In vitro*, hiPSC-ECs responded to flow stress by acquiring an alignment orientation, and attached to and proliferated on the acellular kidney sections, maintaining their phenotype. The hiPSC-ECs were able to self-organize into chimeric kidney organoids to form vessel-like structures. *Ex vivo* infusion of hiPSC-ECs through the renal artery and vein of acellular kidneys resulted in the uniform distribution of the cells in all the vasculature compartments, from glomerular capillaries to peritubular capillaries and small vessels. Ultrastructural analysis of repopulated scaffolds through transmission and scanning electron microscopy demonstrated the presence of continuously distributed cells along the vessel wall, which was also confirmed by 3D reconstruction of z-stack images showing the continuity of endothelial cell coverage inside the vessels. Notably, the detection of fenestrae in the endothelium of glomerular capillaries but not in the vascular capillaries was clear evidence of site-specific endothelial cell specialisation.

## Introduction

Chronic kidney disease (CKD) is a global problem – more than half a million patients reach end-stage renal disease (ESRD) every year, and it causes over 700.000 deaths^1^. Renal replacement therapy (RRT) through dialysis and/or transplantation is currently the only therapeutic option for ESRD patients. However, the shortage of organs available for transplantation poses a major challenge. Moreover, despite advances in immunosuppressive treatments, drug-specific side effects negatively impact allograft function and long-term outcomes. Indeed, the half-life of a transplanted kidney has only changed marginally over the past 20 years, increasing from 9.1 years in 1991 to 10.9 years in 2010^2^, often making a second transplant necessary. Regenerative medicine has exhibited the potential to increase the number of donor organs for transplantation purposes by generating bioengineered organs^3,4^. These strategies are based on the use of natural organ scaffolds and their repopulation with appropriate cells.

In the last few years major efforts have been made to optimize techniques to generate biological scaffolds, which make it possible to maintain the native composition of the extracellular matrix (ECM) and preserve the appropriate three-dimensional (3D) architecture and regional-specific cues needed for cellular adhesion. The first attempt to repopulate orthotopically transplanted acellular scaffolds by homing recipient cells failed, and the implantation of acellular scaffolds was strongly thrombogenic^5^. Indeed, inflammatory cells massively infiltrated the scaffold a week after implantation, inducing severe obstruction of the vasculature by entrapped red blood cells. However, while inflammation was partially reabsorbed three weeks after implantation, repopulation was never observed, in the face of an initial degradation of the ECM, making *ex vivo* reseeding of the acellular organ before implantation imperative^5^. Similar observations had been made with porcine kidney and liver scaffolds^6,7^ and with rat and human lungs^8^, suggesting that the re-establishment of endothelial coverage is absolutely necessary. An initial attempt in this regard had been made by using undifferentiated mouse embryonic stem cells (mES), due to their ability to undergo differentiation as a response to stimuli from cell contact with the underlying ECM matrix. Pioneering studies by Ross *et al*. showed that mES injected into the renal artery (RA) of an acellular rat kidney were able to adhere to vascular structures and were induced to differentiate toward the endothelial lineage^9,10^. A study by our group confirmed these observations, showing that 24 hours after infusion through the renal artery, mES could be detected in the vascular network as far as the glomerular capillaries. Notably, the 3D-ECM induced cells to acquire the expression of mesoderm-derived endothelial lineage markers Tie-2 and CD31 from 24 to 72 hours after cell perfusion, concomitant with a reduction in the pluripotency marker Oct4^11^. To avoid the *in vivo* differentiation process, which could be incomplete – leaving some mES cells undifferentiated – and promote rapid and functional re-endothelialization of the scaffolds, other groups used mature cells, including HUVEC^12^ or mouse pancreatic endothelial cells^13^, which yielded encouraging results in terms of the degree of scaffold repopulation, reaching approximately 70% of cellular glomeruli per regenerated kidney^12^. The main challenge to translating such an approach into clinical practice is the need to achieve a higher percentage of vascular re-endothelialization, using clinically appropriate cell types, overcoming the obstacles to uniform cell seeding previously observed due to the leakage of the scaffold ECM itself^5^.

Induced pluripotent stem cells are the ideal cell source for many biomedical applications, including tissue engineering. By virtue of their capacity for self-renewal and pluripotent differentiation, these cells can be used to generate progenitors and mature cells to repopulate the organ scaffolds and, ideally, regenerate a patient-specific organ. In this study, we first derived endothelial cells from human pluripotent stem cells (iPSC-derived ECs). After phenotypic and functional characterization *in vitro* and *ex vivo*, we studied their ability to repopulate acellular rat kidney scaffolds.

## Results

### Generation and characterization of human iPSC-derived endothelial cells

**Figure 1:**
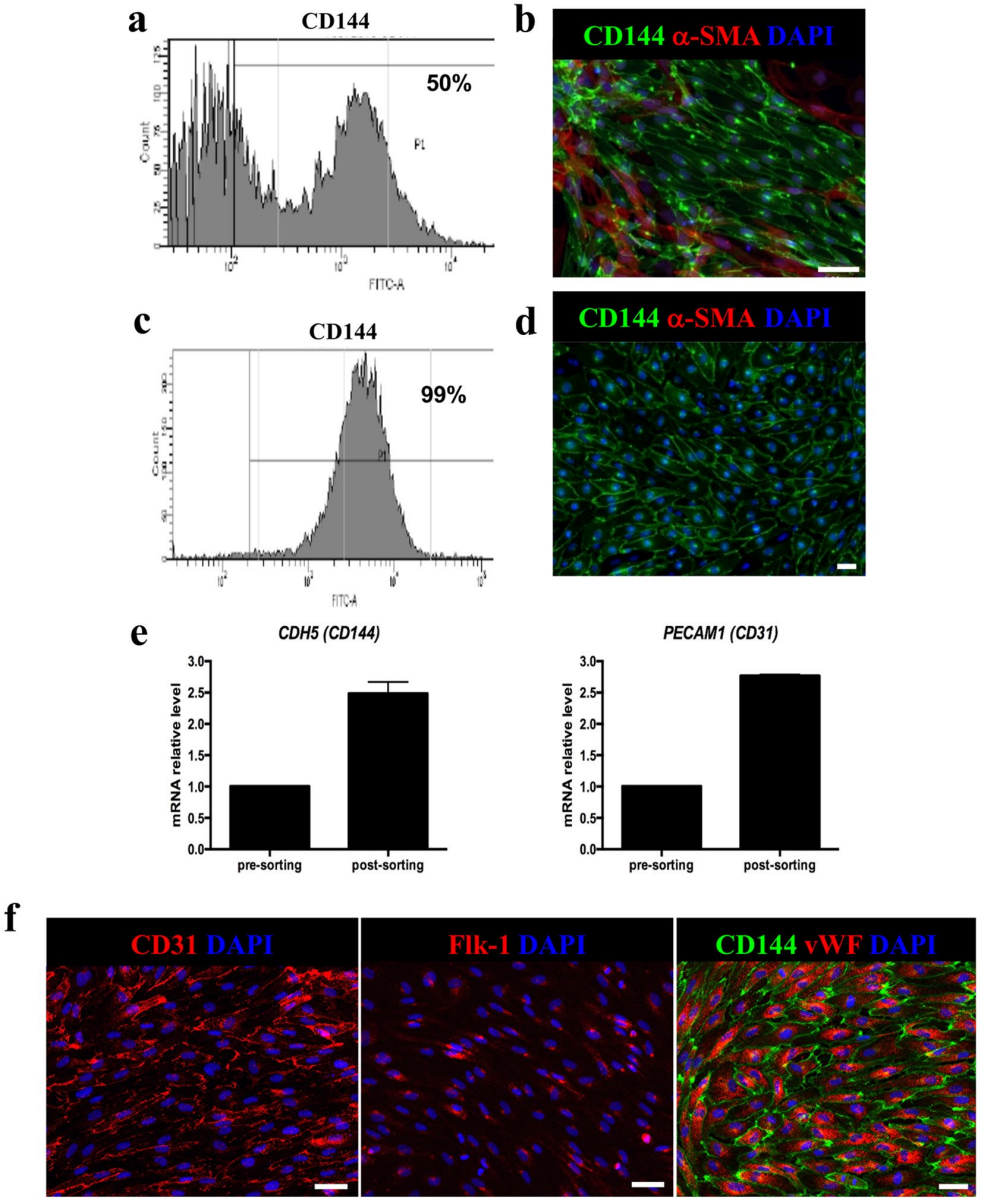
iPSC- derived ECs characterization. (**a**) Representative cytofluorometric analysis of CD144 expression at day 6 of differentiation; (**b**) Representative image of CD144 and α-SMA staining at day 6 of differentiation. Scale bar 50 μm; (**c**) Representative cytofluorometric analysis of CD144 expression 48 h after MACS sorting; (**d**) Representative image of CD144 and α-SMA staining 48 h after MACS separation; (**e**) CD144 and CD31 gene expression analysis in pre- and post-sorted iPSC- derived ECs. Data are expressed as mean ± SD; (**f**) Representative images of CD31, Flk-1 and CD144 and vWF staining. Scale bar 50 μm.

We tested a recently published protocol^14^ for its efficiency and robustness in generating endothelial progenitor cells from human iPS cell lines. The protocol very rapidly and efficiently induced the differentiation of iPSCs toward endothelial progenitors – within six days. One day after seeding, cells were exposed to induction medium for commitment toward the mesodermal fate. Gene expression analysis and immunofluorescence experiments showed a progressive time-dependent decline in the pluripotency markers *OCT4* and *NANOG*, and transient expression of *T-Brachyury (T-BRY)*, peaking between days 2 and 3, which confirmed that cells were acquiring a posterior primitive streak-like phenotype (Supplementary Fig. S1a,b). The endothelial phenotype was achieved by exposing cells for 2 days to VEGF-A and forskolin, a potent activator of adenylyl cyclase, via the activation of protein kinase-A. The identity of the differentiated cells was evaluated by analysing the expression of vascular endothelial cadherin, VE-cadherin and CD144. Flow cytometric analysis revealed that up to 50% of cells were positive for the endothelial marker CD144 (50.85 ± 6.99 n = 3, Fig. 1a). This data was in line with previously reported data that described the generation of a mixed cell population, which included both CD144 and perivascular α-smooth muscle actin (α-SMA) positive cells^14^ as well as being confirmed by immunofluorescence analysis for both CD144 and α-SMA (Fig. 1b). To obtain a pure cell population, CD144^+^ cells were isolated through magnetic-associated cell sorting (MACS). After selection, up to 99% of cells were CD144^+^ and α-SMA^−^ (98.3 ± 1 n = 3, Fig. 1c) as shown by FACS analysis, immunostaining (Fig. 1d) and by the increased gene expression for CD144 and CD31 in post-sorted cells (Fig. 1e). With a view to clinical application, which would require large-scale *ex vivo* expansion, we defined the conditions necessary to promote the expansion and long-term maintenance of EC cells. To this end, purified hiPSC-derived CD144^+^ ECs were plated on human fibronectin-coated plates in EGM-2 medium supplemented with 20% FBS^15^ in the presence of SB431542, a TGF-β inhibitor known to support endothelial cell expansion for up to ten cell divisions^16^. This medium enabled the expansion of one million CD144^+^ ECs, up to over two-hundred million cells in only two weeks. After expansion, cells (cultured in maintenance medium) homogeneously expressed the endothelial markers CD31 as well as Flk-1 – a vascular endothelial growth factor receptor 2 (VEGF-R2) that is critical for the formation and maintenance of endothelial phenotype – and vWF. CD31 was expressed on the cellular membrane borders, while the vWF was markedly expressed with a punctuate pattern in the cytoplasm of CD144-positive cells (Fig. 1f).

### Human iPSC-derived ECs respond to 2D fluid shear stress

**Figure 2:**
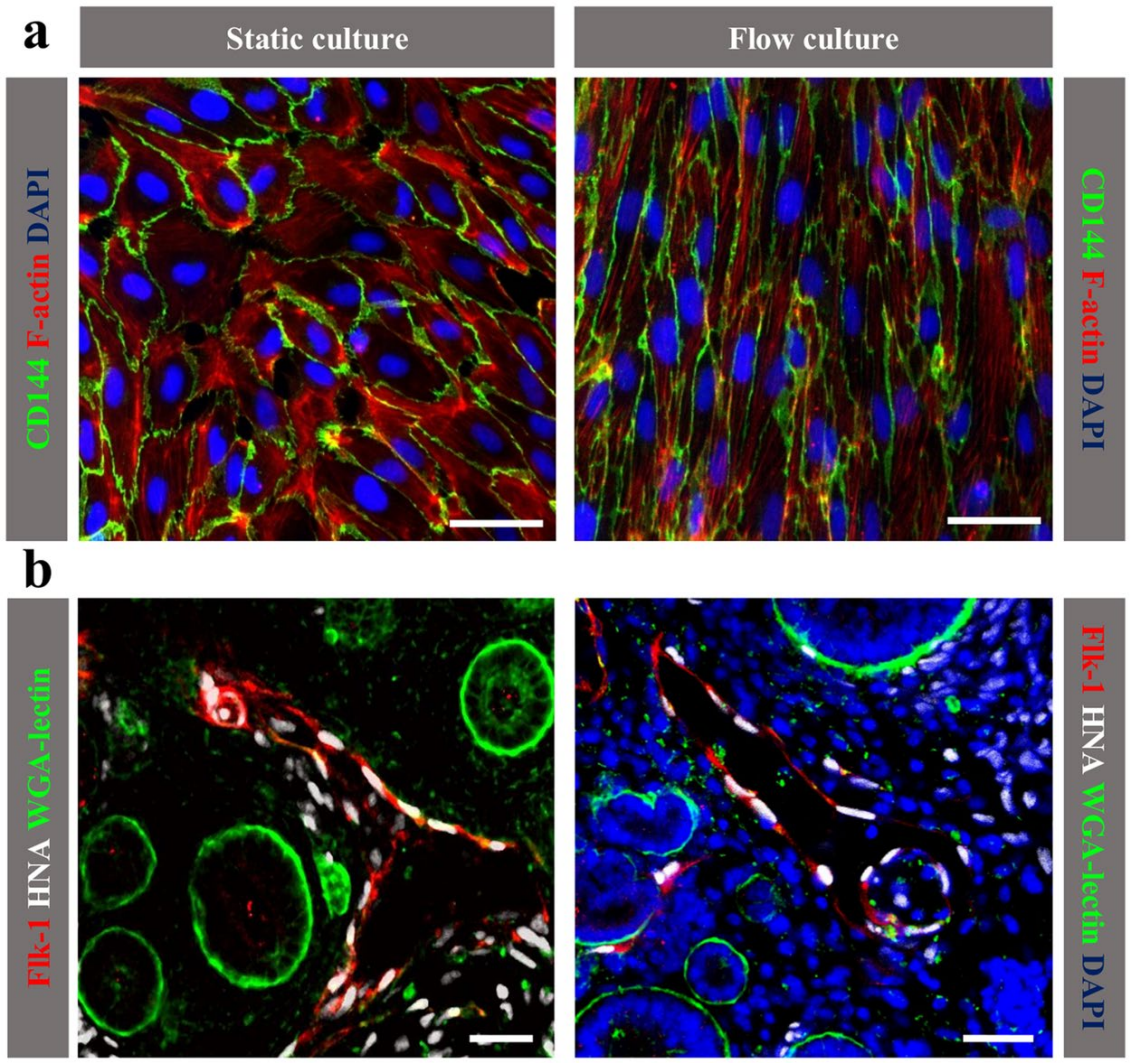
iPSC-derived ECs functionality. (**a**) Immunofluorescence analysis of CD144 and F-actin shows the morphology of iPSC-derived ECs under fluid shear stress (right panel) compared to static culture (left panel). Scale bar 50 μm; (**b**) Integration of human iPSC-derived ECs into the developing chimeric kidney organoid. iPSC-derived ECs (white) express Flk-1 (red). Renal structures were labelled by WGA lectin (green). Scale bars: 50 μm (left panel), 20 μm (right panel).

To maintain vascular homeostasis, endothelial cells respond to physiologic and pathologic hemodynamic forces by undergoing dynamic structural adaptation. *In vitro* studies on HUVEC, by others and by us, have shown that endothelial cells exposed to different shear stress waveforms respond by modifying their shape and function as a consequence of the dynamic regulation of networks of transcription factors and genes^17,18^. Here, we studied the capacity of iPSC-derived endothelial cells to respond to shear stress after exposure to a low and steady flow rate of 3 dynes/cm^2^ for 12 hours. As previously observed with HUVEC, iPSC-derived ECs exposed to unidirectional flow assumed an elongated shape in the direction of the flow, with well-organized distribution of elongated F-actin fibres across the cell body, compared to cells maintained under static conditions, which exhibited a random arrangement of the fibres, mostly located at the cell periphery (Fig. 2a).

### Human iPSC-derived ECs display vasculogenic abilities in kidney organoids

To evaluate the ability of iPSC-derived ECs to form vessels in the 3D tissue environment, we generated kidney organoids from human ECs and mouse kidney cells and studied vasculogenesis *ex vivo*. To this end, CD144^+^ ECs were mixed with embryonic day (E) 12.5 CD1 kidney cells^19,20^ and cultured as organoids *in vitro* for 5 days. To study the distribution of human iPSC-derived ECs in the kidney organoids, we immunostained with the human nuclear antigen (HNA, white), a human-specific marker, and Flk-1 (red), an endothelial-specific marker. Confocal images revealed that most iPSC-derived ECs formed interstitial vessels (Fig. 2b) among the developing renal tubules. These data indicate that human iPSC-derived ECs can properly integrate into the developing mouse kidney. Some single cells negative for Flk-1 were localized in interstitial spaces but did not affect kidney development.

### Decellularization and recellularization of the vasculature of acellular rat kidney scaffolds

**Figure 3:**
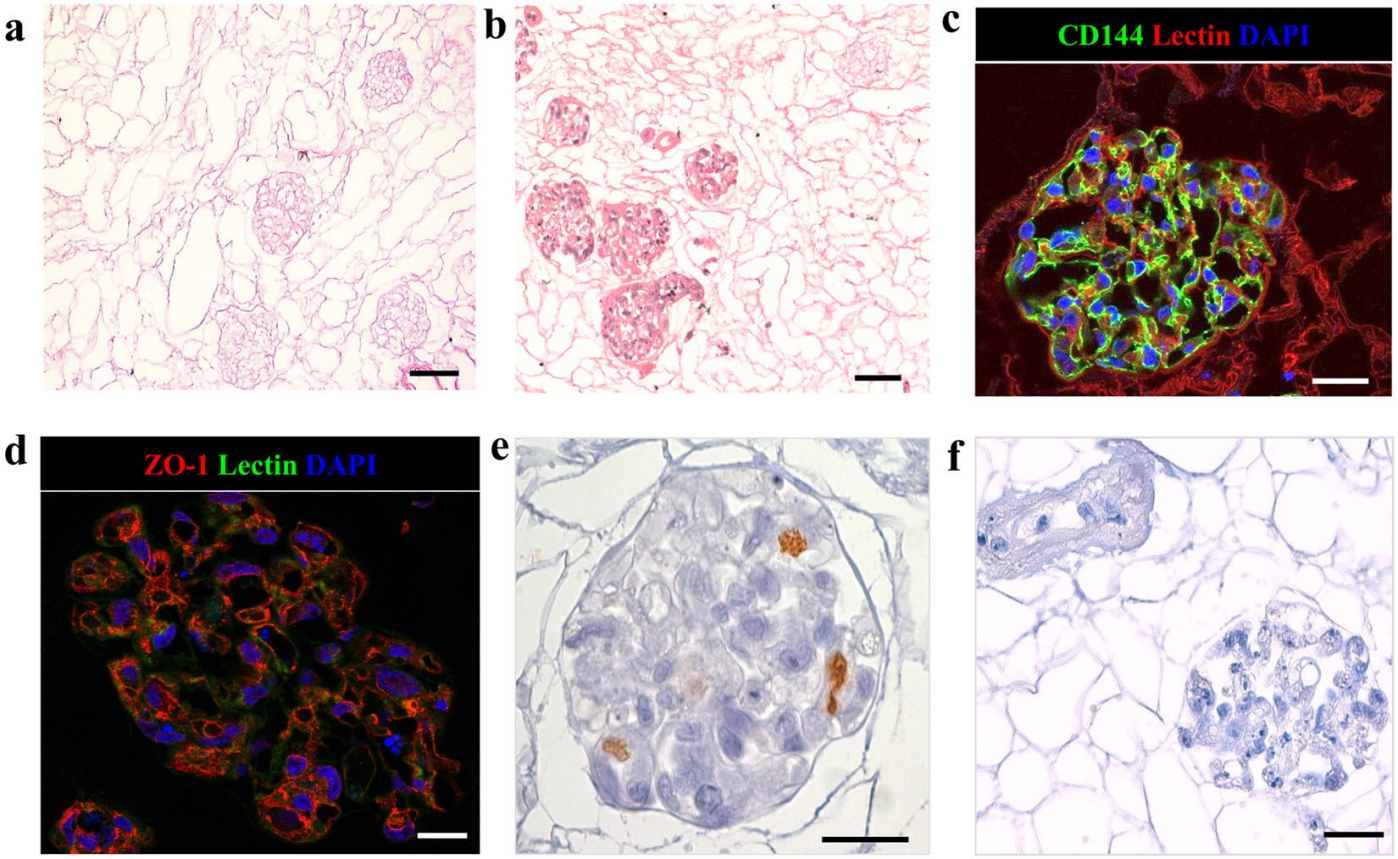
Repopulation of kidney scaffold with iPSC-derived ECs delivered by renal artery. (**a**) Hematoxylin and eosin staining on decellularized kidney scaffold. Scale bar 50 μm; (**b**) Hematoxylin and eosin staining shows cellular distribution mainly in glomerular structures. Scale bar 50 μm; (**c**) Immunofluorescence for CD144 showing iPSC-derived ECs in the glomerular capillary. Scale bar 25 μm; (**d**) ZO-1 staining. Scale bar 20 μm; (**e**) Ki67 staining. Scale bar 20 μm; (**f**) Isotype antibody staining used as negative control. Scale bar 50 μm.

**Figure 4:**
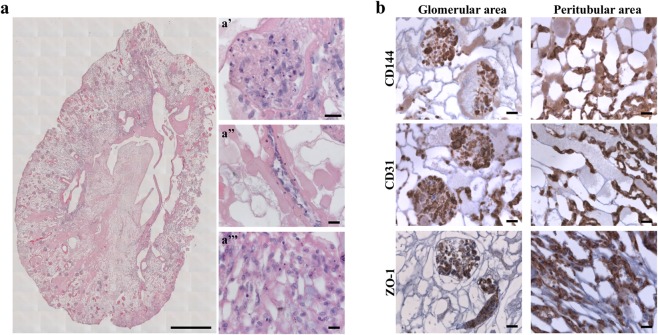
Reseeding of kidney scaffold with iPSC-derived ECs delivered by renal artery and vein. (**a**) Mosaic view of a transversal cross-section of repopulated kidney demonstrating a homogeneous distribution of iPSC-derived ECs into glomeruli and vascular structures. Scale bar 1 cm. (a’ - a”’) Selected images showing iPSC-derived ECs localization into glomerulus (a’), vascular network (a”), peritubular capillaries (a”’). Scale bar 20 μm. (**b**) Characterization of repopulated scaffold by CD144, CD31 and ZO-1 staining shows that iPSC-derived ECs maintain their phenotype into glomeruli and vascular structures. Scale bar 20 μm.

We recently succeeded in obtaining acellular renal scaffolds from rats through detergent perfusion^5,11^. The decellularized kidney scaffolds, examined histologically through hematoxylin and eosin staining and scanning electron microscopy (SEM; Supplementary Fig. S2), demonstrated the absence of cell nuclei and well-preserved vessel architecture, glomeruli and tubules (Fig. 3a). Using the cell seeding technique developed in our laboratories^5^, we attempted to promote re-endothelialization of an acellular rat kidney. In a preliminary experiment, 15 × 10^6^ iPSC-derived CD144^+^ ECs were infused into the renal artery. No macroscopic tissue damage or leakage of cells was observed during the injection procedure. After seeding, kidneys were transferred into the incubator and perfused for 48 hours with recirculating medium. Histological examination with hematoxylin and eosin staining of kidney sections showed that cells had primarily reached the cortical region of the kidney and were distributed in the arterial structures and in the glomerular capillaries of approximately 86% of glomeruli (Fig. 3b). Seeded cells maintained the expression of endothelial marker CD144. Notably, the CD144 peripheral localization on the membrane, both in the glomerulus and in the peritubular capillary, showed ECs forming a continuous network (Fig. 3c), as confirmed by the expression of ZO-1, a component of the tight junctions^8^ (Fig. 3d). Moreover, some cells maintain their proliferation state, as detected by staining with the proliferation marker Ki67 (Fig. 3e and Supplementary Fig. S3). As is evident in Supplementary Fig. S4, not all glomeruli reached by the cells were fully repopulated. ZO-1 staining, used to quantify the percentage of repopulated glomeruli, showed that approximately 40% of the glomeruli reached by the cells were partially repopulated. No staining was detected using an isotype antibody as a negative control (Fig. 3f). In an effort to improve the number of fully repopulated glomeruli and to home the cells in the venous compartment, we increased the number of endothelial cells infused into the renal artery (30 × 10^6^ iPSC-derived CD144^+^ ECs) and performed a second injection of the 30 × 10^6^ iPSC-derived CD144^+^ ECs through the renal vein (RV) for a total of 60 × 10^6^ iPSC-derived CD144^+^ ECs per renal scaffold. The seeded cells were allowed to attach for 2 hours before starting 48 hours of medium recirculation at a maximum flow rate of 1.6 ml/min. The combination of the two routes of cell administration with static flow before perfusion resulted in the widespread distribution of cells in both the cortical and medullar vascular compartment, as detected by hematoxylin and eosin staining (Fig. 4a and Supplementary Fig. S5). This approach to cell infusion allowed the repopulation of glomeruli (Fig. 4a, a’), and of the vascular network (Fig. 4a, a”), including a massive repopulation of the peritubular capillaries (Fig. 4a, a”’). Cells maintained their identity as positive for CD144, CD31 and ZO-1, both in the glomerular and peritubular areas (Fig. 4b). ZO-1 quantification indicated that 89.22 ± 5.04% of glomeruli were reached by iPSC-derived ECs, of which only 13.49 ± 3.37% were partially repopulated.

**Figure 5:**
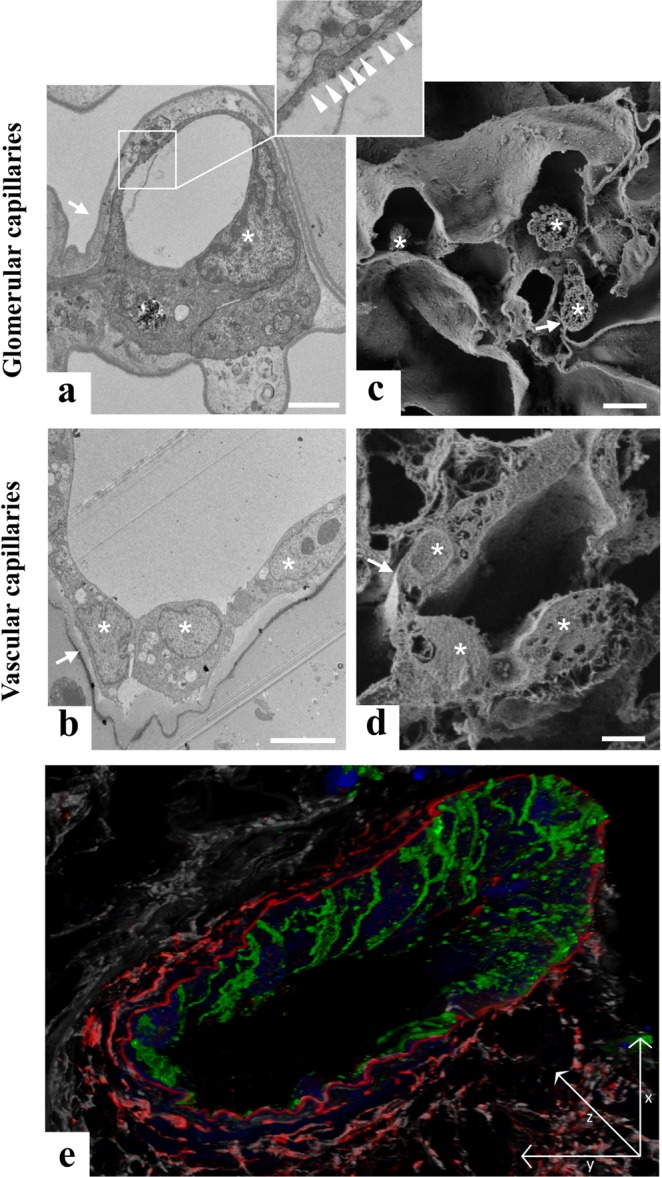
Characterization of iPSC-derived ECs repopulation by electron microscopy and confocal analysis. (**a**) Transmission electron microscopy image of glomerular capillaries showing repopulation by human iPSC-derived ECs, with the corresponding high magnification inset showing a fenestrated endothelium indicated by white arrowheads. Scale bar 2 μm; (**b**) TEM analysis of small arterioles ultrastructure showing cell-to-cell contact between the endothelial cells. Scale bar 5 μm; (**c**) Scanning electron microscopy image of glomerular capillaries. Scale bar 2 μm; (**d**) Scanning electron microscopy image of vascular capillaries. Scale bar 5 μm. In TEM and SEM images, capillary basement membranes and vessel walls are indicated by arrows and endothelial cells are indicated by asterisks; (**e**) Z sectioning and 3D reconstruction show a continuous layer of CD144^+^ ECs (green) in the vessel wall labelled with elastin antibody (red). LCA lectin (white) stains renal structures, DAPI (blue) stains nuclei. Scale bar 25 μm.

Ultrastructural examination through transmission electron microscopy (TEM) allowed us to identify endothelial cells integrated within the vascular network of the glomerular capillary adhering to the vascular lumen (Fig. 5a). A careful and detailed analysis at higher magnification of the glomerular capillary endothelium allowed us to detect the presence of fenestrated endothelium (Fig. 5a, inset). Notably, cross-sections of small arterioles showed cell-to-cell contact between the endothelial cells that formed a continuous monolayer all around the vessel wall (Fig. 5b). Fenestrated endothelium was not detected in the vascular capillary, which is clear evidence of site-specific specialisation of the integrated endothelial cells. Three-dimensional analysis with SEM confirmed the re-cellularization observed with TEM, both in the glomerular capillaries (Fig. 5c) and in small arterioles (Fig. 5d) Finally, a 3D reconstruction of z-stack images of a blood vessel from a 25 µm section of reseeded scaffolds further demonstrated the continuity of endothelial cell coverage inside the vessels (Fig. 5e). Together, TEM and SEM analysis and 3D confocal reconstruction clearly demonstrated the formation of the inner wall of the vessel.

## Discussion

The generation of bio-artificial organs is the most promising strategy for overcoming the gap between the large number of patients waiting for transplantation and the insufficient number of donor organs available. Recent advances in tissue engineering technologies have led to the generation of simple structures, including blood vessels^21^, or bladders^22^, which require a thin monolayer of cells seeded onto a biodegradable scaffold. The more complex the organ to be engineered is, the more complicated its repopulation, and this is particularly true for organs with intricate architecture, like the kidney, which includes more than 26 different cell types^23^. The preservation of an ECM scaffold with a complete vasculature, able to provide nutrients and support seeded-cell viability, is essential for obtaining a bio-artificial kidney. Although it was initially postulated that acellular scaffolds could be replenished after transplantation by host cells, progressive proteolysis, with reabsorption of the scaffold ECM by inflammatory cells was indeed observed^5^, making cellular replenishment of the scaffold before transplantation absolutely necessary. Our group, among others, has successfully reseeded a well-preserved acellular rat kidney scaffold using mouse embryonic stem cells^10,11^. In that study, Bonandrini and co-workers optimized the protocol to generate a 3D whole-kidney scaffold with a well-preserved ECM structure that promoted mouse embryonic stem cells’ acquisition of the same markers as endothelial precursors. These results paved the way for the use of mature endothelial cells ready to properly function immediately after seeding.

In this study, we have demonstrated that endothelial cells generated from iPSCs are a valuable cell source for replenishing the renal vascular bed. Firstly, these cells can easily be expanded *in vitro*, which makes it possible to translate the procedure into clinical practice. They express the specific markers and share the typical features of their *in vivo* counterparts, including the ability to respond to stimuli like shear stress and to properly integrate into the developing kidney to form vascular structures. When infused through the renal artery and vein, endothelial cells reached all vascular compartments, allowing the repopulation of 89% of the glomeruli. Cells were also well distributed in the peritubular capillaries, from the cortical to the medullar portion of the renal section, and in the small arterioles where a monolayer of cells covered the inner wall of the vessel.

The use of iPSC-derived endothelial cells to repopulate the renal scaffold is a step forward compared to previous studies. The first studies to attempt organ engineering seeded the scaffolds with primary cells or cell lines. Song and co-workers infused HUVEC through the renal artery, demonstrating cell seeding in 70% of glomeruli^12^. The injection of neonatal rat kidney cells through the ureter allowed them to reach the tubular compartment, generating a very rudimentary kidney. While HUVEC may prove that this approach is feasible, the use of patient-derived cells to repopulate the scaffold would allow for the generation of personalized organs, as was attempted for the lung, where endothelial cells obtained from human iPSCs were used to repopulate decellularized rat lungs, reaching about 75% endothelial coverage^8^.

The difficulty with establishing vasculature with sufficient endothelial cell coverage is one of the biggest obstacles to achieving transplantable epithelial organs. For the kidney, the regeneration of the glomerulus is the most difficult step in establishing functional renal tissue. A first attempt to repopulate a kidney scaffold using iPSC-derived endothelial cells has shown that endothelial cells improve the differentiation of co-injected renal epithelial progenitors^24^. However, this study neglected to clearly show the endothelial regeneration of the glomerular and peritubular capillaries by endothelial cells, and renal tubules were rarely repopulated by epithelial cells. These limitations were probably due to the fact that endothelial and epithelial cells were injected simultaneously, which did not allow endothelial cells to properly attach to the basement membranes and epithelial cells to reach the appropriate compartments unless there was a rupture of the glomerular basement membrane during the injection procedure^24^, which is very likely, since cells were perfused manually into the scaffold. This point has been discussed in depth by Remuzzi *et al*.^5^. Indeed, although high pressure cell injection induced broad distribution of the cells into the scaffold, this result was actually a consequence of extravasation and the accumulation of the cells following capillary and tubular wall rupture induced by high pressure.

To overcome the hurdles to achieving more uniform and extensive distribution of the cells along the entire acellular vasculature, in the current study we perfused a high number of endothelial cells under controlled flow rate, both through the renal artery and through the renal vein. This perfusion protocol allowed the cells to reach not only the arterial vessels up to the glomerular capillaries but also to reach the renal veins and peritubular capillaries. The increased number of cells injected through the renal artery allowed us to reduce the percentage of partially repopulated glomeruli from 40 to 13.49%. Electron microscopy images of the seeded scaffolds clearly highlighted the presence of endothelial cells within the glomerular capillaries adhering to the vascular lumen. What surprised us most was that only two days after injection, the adherent endothelial cells had specialised by generating a fenestrated endothelium in the glomerular capillaries but not in the vascular capillaries. The presence of fenestrae in the flat region of the endothelial cells at the capillary loop periphery is indispensable for the hydraulic conductivity of the glomerular capillary wall and is clear evidence of a specialised endothelium^25,26^. The endothelial cells were also observed in small arterioles, where ultrastructural analysis and 3D reconstruction of the blood vessel showed the presence of strings of endothelial cells with normal morphology. With our technique, we improved the endothelial coverage of kidney scaffolds, an essential prerequisite for a more complex repopulation procedure, which involves accommodating other cell types in the scaffold, in addition to the ones generated *in vitro* by our recently published protocol demonstrating the differentiation of human iPSC toward renal progenitor cells^27^.

In conclusion, here we have characterized iPSC-derived endothelial cells in terms of response to shear stress and ability to properly integrate into embryonic kidney structures forming vascular structure. We demonstrated how to efficiently engineer the vasculature of renal scaffolds, showing for the first time that human iPSC-derived endothelial cells were able to cover 89% of the glomeruli and extensively repopulate peritubular capillaries. In spite of these promising results, several issues still need to be addressed before this approach can be translated into clinical practice. Combining endothelial coverage with the repopulation of the glomerular and tubular compartment by infusing a population of renal progenitor cells will make it possible to generate a kidney, whose function will be properly evaluated after orthotopic transplantation in syngeneic rats. These results could possibly pave the way for the regeneration of a patient-specific kidney, starting from a whole-kidney scaffold harvested from a patient with progressive CKD, a strategy that could fill the gap between the unsustainable costs of dialysis treatment, the organ donor shortage and the side effects of antirejection drugs.

## Methods

### Human iPSC culture

The hiPSC Clone IV cell lines generated in our laboratories^28^ were cultured on hESC-qualified matrigel-(BD Biosciences) coated dishes in mTeSR1 medium (StemCell Technologies) and split twice a week using Accutase (ThermoFisher Scientific).

### Human iPSC differentiation into endothelial cells

To obtain endothelial cells, hiPSC Clone IV was exposed to a differentiation protocol, as previously described by Patsch^14^, with few modifications. Detailed protocol is described in Supplementary Methods. For expansion, CD144^+^ cells were maintained in EGM-2 medium (Lonza) without hydrocortisone, supplemented with 20% defined FBS (Hyclone) and 10 μM SB431542. All the experiments described in the manuscript were performed with hiPSC-derived ECs between passages three and five.

### Flow cytofluorometric analysis

Evaluation of CD144 expression was performed by flow cytometer FACs Canto (BD Bioscience). Cells were fixed with 2% paraformaldehyde (PFA) for 15 min on ice and then incubated for 30 min with 1% BSA. Cells were stained for 1 h with CD144 primary antibody (R&D System) diluted according to the manufacturer’s recommendations followed by the appropriate secondary antibody. Samples treated with secondary antibody alone were used as controls.

### Immunofluorescence on cells

The cells were fixed with 4% PFA for 15 min at room temperature (RT), permeabilized with 0.3% Triton X-100 (Sigma) for 15 min at RT (when necessary) and then incubated for one hour with 5% BSA as a blocking solution. The samples were stained with the primary antibody diluted in 2% BSA overnight at 4 °C followed by incubation with the appropriate secondary antibody for 1 h at RT. Nuclei were counterstained with 4’,6-diamidino-2-phenylindole (DAPI) for 5 minutes at RT. Images were taken using fluorescence microscope (Apotome Axio Imager Z2, Zeiss) and are representative of n = 3 experiments. Primary antibodies are listed in Supplementary Information Table S1.

### Immunofluorescence on tissue

Five μm-thick periodate-lysineparaformaldehyde (PLP)–fixed cryosections were incubated with 5% BSA and then with goat anti-CD144 (1:100) or rabbit anti-ZO-1 (ThermoFisher Scientific, 1:200) followed by the appropriate secondary antibody (ThermoFisher Scientific). Nuclei were labelled with DAPI and renal structures with rhodamine lens culinaris agglutinin or fluorescein wheat germ agglutinin (Vector Laboratories). Negative controls were obtained by omitting the primary antibody on adjacent sections. For the 3D reconstruction of vessel walls, immunofluorescence analysis was performed on 25 μm-thick Duboscq-Brazil-fixed, paraffin-embedded renal sections. Antigen retrieval was performed using a Decloaking chamber (DC NxGen 220 V, Biocare Medical) for 15 minutes at 110 °C with DIVA decloaker buffer (Biocare Medical). After blocking with 3% BSA, sections were incubated with goat anti-CD144 (1:100) and rabbit anti-elastin (Abcam, 1:100), followed by the appropriate secondary antibodies. Renal structures were labelled with DyLight 649 lens culinaris agglutinin (Vector Laboratories) and nuclei with DAPI. Fluorescence was examined using an inverted confocal laser-scanning microscope (Leica TCS SP8, Leica microsystems).

### Immunoperoxidase experiments

Duboscq-Brazil-fixed, paraffin-embedded renal sections (3 μm) were deparaffinized and incubated with Peroxidazed1 solution (Biocare Medical) to quench endogenous peroxidases. Antigen retrieval was performed as described above. After blocking with Background Punisher (Biocare Medical), sections were incubated with rabbit anti-Ki67 (Abcam), goat anti-CD144 (1:100), rabbit anti-CD31 (1:150), or rabbit anti-ZO-1 (1:200), followed by MACH4 or goat-on-rodent-HRP Polymer kit (Biocare Medical) and diaminobenzidine substrate solution. Slides were finally counterstained with Meyer’s hematoxylin, dehydrated in graded alcohols and observed by light microscopy (Apotome Axio Imager Z2, Zeiss). Isotype control staining was used to exclude the nonspecific binding (normal rabbit IgG, Santa Cruz Biotechnology). Glomerular repopulation was quantified as the percentage of glomeruli reached by iPSC-derived ECs of the total number of glomeruli observed (100%). At least 1200 glomeruli were examined on ZO-1-stained sections. Glomeruli were considered fully repopulated when the iPSC-derived EC repopulation involved 75% to 100% of the glomerular tuft. When the repopulation involved less than 75% of the glomerular area, glomeruli were considered partially repopulated. Data are expressed as mean ± SD.

### Gene expression analysis

Total RNA was isolated with Trizol and treated with DNAse (Promega) as described by the manufacturer. cDNA was obtained using Superscript VILO cDNA Synthesis Kit (ThermoFisher Scientific). Quantitative real-time PCR assays were performed using Taqman gene expression assays (ThermoFisher Scientific) and predesigned Taqman probes (Supplementary Information Table S2). Gene expression levels were normalized to the housekeeping gene *HPRT1*.

### 2D fluid shear stress experiment

To investigate the response to shear stress, iPSC-derived EC monolayers were cultured under static and flow conditions. The parallel plate flow chamber used to expose the cell monolayer to laminar flow conditions has already been described^29^. The hiPSC-derived ECs were seeded at a density of 28.000 cells/cm^2^ on 5 μg/cm^2^ fibronectin-coated glass and cultured in maintenance medium for 4 days, changing the medium every other day. Prior to flow initiation, the culture medium was exchanged for fresh growth medium supplemented with 4% dextran (Sigma). The same 4% dextran-supplemented medium was used for the control static condition. Medium flow rate was gradually increased over a 10-min period, to expose the cell monolayer to a wall shear stress value up to 3 dynes/cm^2^, then the flow rate was kept constant until the end of the experiment (12 hours). Finally, cells were processed for immunofluorescence using goat anti-CD144, rhodamine phalloidin and DAPI, respectively.

### Kidney organoid cultures and immunofluorescence analysis

Kidney organoids were constructed and cultured as previously described^19,30^. Briefly, E12.5 CD1 mouse (Charles River Italia SpA) kidneys were dissected in MEM (Sigma), and dissociated into single cell suspensions. The mouse cells were centrifuged in the presence of hiPSC-derived ECs at a ratio of 10:1 (mouse/human). Kidney organoid aggregates were cultured at the air-medium interface in Advanced DMEM (ThermoFisher Scientific) supplemented with 2% Embryonic Stem Cell Fetal Bovine Serum (ES-FBS, Gibco), 1% L-glutamine, 1% penicillin/streptomycin and 1.25 μM Glycyl-H1152 dihydrochloride (Tocris) only for the first 24 hours. At 5 days, kidney organoids were fixed in 4% PFA for 10 min, permeabilized with 100% cold methanol for 10 min, and incubated with mouse anti-Human Nuclear Antigen (HNA, 1:50; Merck Millipore Ltd), rabbit anti-Flk-1 (1:50) antibodies, followed by the specific secondary antibody. Renal structures were labelled with fluorescent Wheat Germ Agglutinin (WGA, 1:400; Vector Laboratories) and nuclei with DAPI. Fluorescence was examined on an inverted confocal laser-scanning microscope (LSM 510 Meta; Zeiss).

### Kidney retrieval and organ decellularization

Adult male Sprague-Dawley rats (Charles River Laboratories International, Inc.), weighing 250–400 g, were used. After anaesthesia, a longitudinal abdominal incision was made and the kidney, aorta, vena cava, and ureter were identified. The RA and RV were cannulated with a PE50 catheter and a 14-gage cannula, respectively, and then secured with a 4/0 silk suture. After blanching, the kidney was harvested and maintained immersed in saline solution until processing. Kidney decellularization was performed as previously described^5,11^. Detailed protocol is reported in Supplementary Methods.

### Recellularization of kidney scaffolds with hiPSC-derived ECs

Before cell seeding, the kidney scaffold was washed in open circuit with 1% (v/v) penicillin/streptomycin in PBS solution for 2 h. Thereafter, the kidney was perfused at 37 °C for 1 h with hiPSC-derived ECs culture medium. For RA endothelial delivery (n = 1), three suspensions containing 5 × 10^6^ cells (15 × 10^6^ cells in total) were infused at 1 ml/min through the arterial cannula connected to a syringe pump (Harvard Apparatus). Each infusion was followed by recirculation of culture medium for 10 minutes at 0.4 ml/min. After cell seeding, the kidney scaffolds were kept in a cell incubator and continuously perfused with recirculating medium through the RA for 48 hours at a flow rate of 0.4 ml/min. For RA and RV endothelial delivery (n = 3), four suspensions containing 7.5 × 10^6^ cells (30 × 10^6^ cells in total) were injected trough RA as described above, followed by the infusion of four suspensions containing 7.5 × 10^6^ cells (30 × 10^6^ cells in total) through RV carried out with the same procedure for arterial infusion. After two hours of static culture, perfusion was initiated at 0.4 ml/min and progressively increased to 1.6 ml/min in the first 24 hours and maintained for an additional 24 hours. After 48 h, the kidneys were harvested, cut into five transverse sections and fixed in Duboscq-Brazil (n = 2), PLP (n = 2) and 2.5% glutaraldehyde (n = 1).

### Transmission electron microscopy (TEM)

Glutaraldehyde-fixed fragments of kidney tissue were washed repeatedly in cacodylate buffer, post-fixed in 1% OsO_4_, dehydrated through ascending grades of alcohol, and embedded in Epon resin. Ultrathin sections were stained with uranyl acetate and lead citrate and examined using a TEM (Morgagni 268D, Philips).

### Scanning electron microscopy (SEM)

*K*idney biopsy specimens were fixed in 2.5% glutaraldehyde, washed in cacodylate buffer and post-fixed in 1% osmium tetroxide. Samples were then dehydrated in graded ethanols, critical point-dried in carbon dioxide and coated with atomic gold particles prior to viewing with a SEM (Supra 55, Zeiss).

### Ethical issues

The Institutional Animal Care and Use Committees of the Istituto di Ricerche Farmacologiche Mario Negri IRCCS, Milan, Italy, approved all animal studies. Animal care and treatment were conducted in accordance with the institutional guidelines, in compliance with national (D.L.n.26, March 4, 2014), and international laws and policies (directive 2010/63/EU on the protection of animals used for scientific purposes). Animals were housed in a constant-temperature room with a 12-hour dark/12-hour light cycle in a specific pathogen-free facility, and fed a standard diet.

## Supplementary information

Supplementary Information

## Data Availability

All data generated or analysed during this study are included in this published article (and its Supplementary Information files).

## References

[1] Lozano R (2012). Global and regional mortality from 235 causes of death for 20 age groups in 1990 and 2010: a systematic analysis for the Global Burden of Disease Study 2010. Lancet.

[2] Lamb KE, Lodhi S, Meier-Kriesche HU (2011). Long-term renal allograft survival in the United States: a critical reappraisal. Am J Transpl..

[3] Moser PT, Ott HC (2014). Recellularization of organs: what is the future for solid organ transplantation?. Curr. Opin. Organ Transplant..

[4] Destefani AC, Sirtoli GM, Nogueira BV (2017). Advances in the Knowledge about Kidney Decellularization and Repopulation. Front. Bioeng. Biotechnol..

[5] Remuzzi A (2017). Experimental Evaluation of Kidney Regeneration by Organ Scaffold Recellularization. Sci. Rep..

[6] Orlando G (2012). Production and implantation of renal extracellular matrix scaffolds from porcine kidneys as a platform for renal bioengineering investigations. Ann Surg.

[7] Ko IK (2015). Bioengineered transplantable porcine livers with re-endothelialized vasculature. Biomaterials.

[8] Ren X (2015). Engineering pulmonary vasculature in decellularized rat and human lungs. Nat Biotechnol.

[9] Ross EA (2009). Embryonic stem cells proliferate and differentiate when seeded into kidney scaffolds. J. Am. Soc. Nephrol. JASN.

[10] Ross EA (2012). Mouse stem cells seeded into decellularized rat kidney scaffolds endothelialize and remodel basement membranes. Organogenesis.

[11] Bonandrini B (2014). Recellularization of well-preserved acellular kidney scaffold using embryonic stem cells. Tissue Eng Part A.

[12] Song JJ (2013). Regeneration and experimental orthotopic transplantation of a bioengineered kidney. Nat Med.

[13] Ko IK (2014). Enhanced re-endothelialization of acellular kidney scaffolds for whole organ engineering via antibody conjugation of vasculatures. Technology.

[14] Patsch C (2015). Generation of vascular endothelial and smooth muscle cells from human pluripotent stem cells. Nat. Cell Biol..

[15] Melero-Martin JM (2007). *In vivo* vasculogenic potential of human blood-derived endothelial progenitor cells. Blood.

[16] James D (2010). Expansion and maintenance of human embryonic stem cell-derived endothelial cells by TGFbeta inhibition is Id1 dependent. Nat Biotechnol.

[17] Ajami NE (2017). Systems biology analysis of longitudinal functional response of endothelial cells to shear stress. Proc. Natl. Acad. Sci. USA.

[18] Franzoni M (2016). Endothelial cell activation by hemodynamic shear stress derived from arteriovenous fistula for hemodialysis access. Am. J. Physiol. Heart Circ. Physiol..

[19] Xinaris C (2012). *In vivo* maturation of functional renal organoids formed from embryonic cell suspensions. J. Am. Soc. Nephrol. JASN.

[20] Xinaris C (2016). Functional Human Podocytes Generated in Organoids from Amniotic Fluid Stem Cells. J. Am. Soc. Nephrol. JASN.

[21] Hibino Narutoshi, McGillicuddy Edward, Matsumura Goki, Ichihara Yuki, Naito Yuji, Breuer Christopher, Shinoka Toshiharu (2010). Late-term results of tissue-engineered vascular grafts in humans. The Journal of Thoracic and Cardiovascular Surgery.

[22] Atala A, Bauer SB, Soker S, Yoo JJ, Retik AB (2006). Tissue-engineered autologous bladders for patients needing cystoplasty. Lancet.

[23] Al-Awqati Q, Oliver JA (2002). Stem cells in the kidney. Kidney Int..

[24] Du C (2016). Functional Kidney Bioengineering with Pluripotent Stem-Cell-Derived Renal Progenitor Cells and Decellularized Kidney Scaffolds. Adv. Healthc. Mater..

[25] Satchell SC, Braet F (2009). Glomerular endothelial cell fenestrations: an integral component of the glomerular filtration barrier. Am. J. Physiol. Renal Physiol..

[26] Obeidat M, Obeidat M, Ballermann BJ (2012). Glomerular endothelium: a porous sieve and formidable barrier. Exp. Cell Res..

[27] Ciampi O (2016). Generation of functional podocytes from human induced pluripotent stem cells. Stem Cell Res.

[28] Imberti B (2015). Renal progenitors derived from human iPSCs engraft and restore function in a mouse model of acute kidney injury. Sci Rep.

[29] Cattaneo I (2011). Shear stress reverses dome formation in confluent renal tubular cells. Cell. Physiol. Biochem. Int. J. Exp. Cell. Physiol. Biochem. Pharmacol..

[30] Benedetti* Valentina, Brizi* Valerio, Xinaris Christodoulos (2016). Generation of Functional Kidney Organoids In Vivo Starting from a Single-Cell Suspension. Methods in Molecular Biology.

