# Supplementary material for: Engineering the vasculature of decellularized rat kidney scaffolds using human induced pluripotent stem cell-derived endothelial cells
Source: Sci Rep. 2019 May 29;9:8001. doi: 10.1038/s41598-019-44393-y (PMC6541625; doi:10.1038/s41598-019-44393-y)
Supplement: Supplementary file 1 — Supplementary Information [file 41598_2019_44393_MOESM1_ESM.pdf]

Engineering the vasculature of decellularized rat kidney scaffolds using human induced pluripotent stem cell-derived endothelial cells

Osele Ciampi[1], Barbara Bonandrini[1,2], Manuela Derosas[1], Sara Conti[1], Paola Rizzo[1], Valentina Benedetti[1], Marina Figliuzzi[1], Andrea Remuzzi[3], Ariela Benigni[1], Giuseppe Remuzzi[1,4,], Susanna Tomasoni[1]

[1]Istituto di Ricerche Farmacologiche Mario Negri IRCCS,
Centro Anna Maria Astori, Science and Technology Park Kilometro Rosso, 24126 Bergamo, Italy
[2]Department of Chemistry, Materials and Chemical Engineering "Giulio Natta",
Politecnico di Milano, 20133 Milan, Italy
[3]Department of Industrial Engineering, Bergamo University, 24044 Dalmine (Bergamo), Italy
[4]'L. Sacco' Department of Biomedical and Clinical Sciences, University of Milan, 20122 Milan, Italy

* Correspondence should be addressed to:

Susanna Tomasoni, PhD

Istituto di Ricerche Farmacologiche Mario Negri IRCCS
Centro Anna Maria Astori
Science and Technology Park Kilometro Rosso
Via Stezzano, 87
24126 Bergamo, Italy
Tel: +39-035-42131; Fax: +39 035-319331
email: susanna.tomasoni@marionegri.it

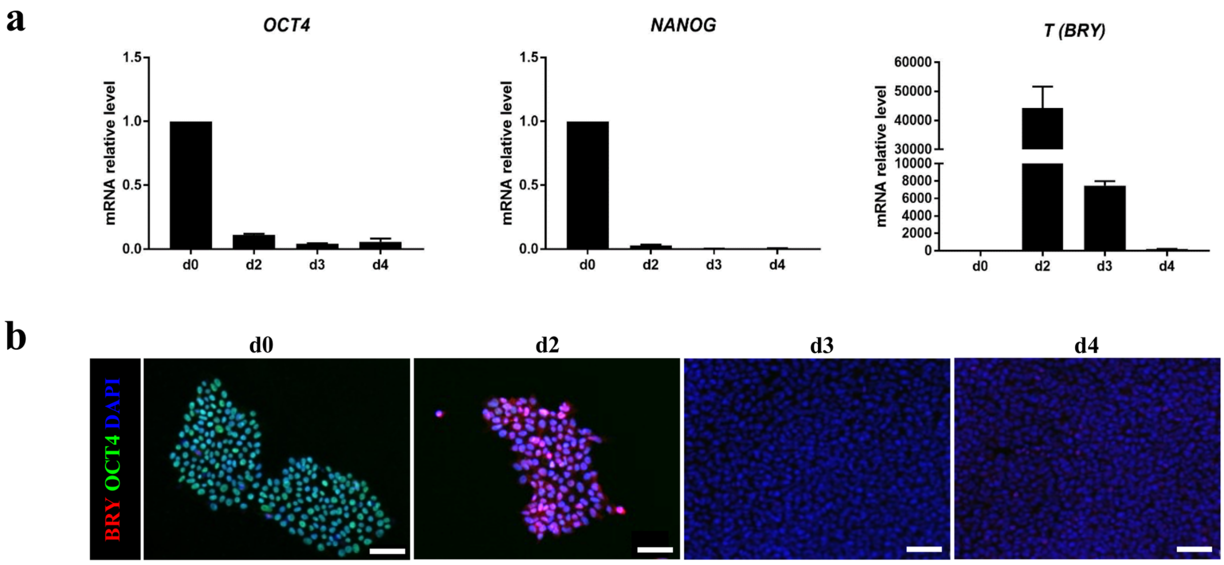

Figure S1. iPSC commitment into mesoderm. (a) Gene expression analysis shows decreased expression of pluripotency marker *OCT4* and *NANOG* and the induction of the mesoderm marker *T/(BRY)* expression. Data are expressed as mean ± SD; (b) Representative images of OCT4 and BRY staining confirm the progressive loss of OCT4 expression and induction towards the mesodermal fate. Scale bar 50 μm.

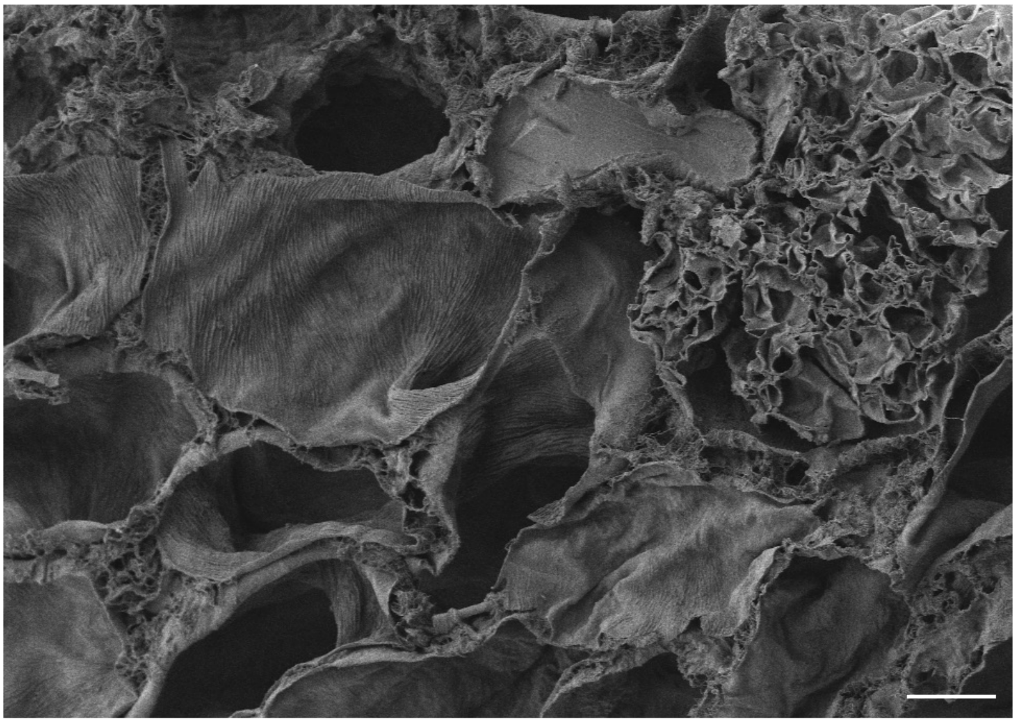

Figure S2. SEM image of decellularized scaffold. Image shows the absence of cells and well-preserved structures. Scale bar 10 μm.

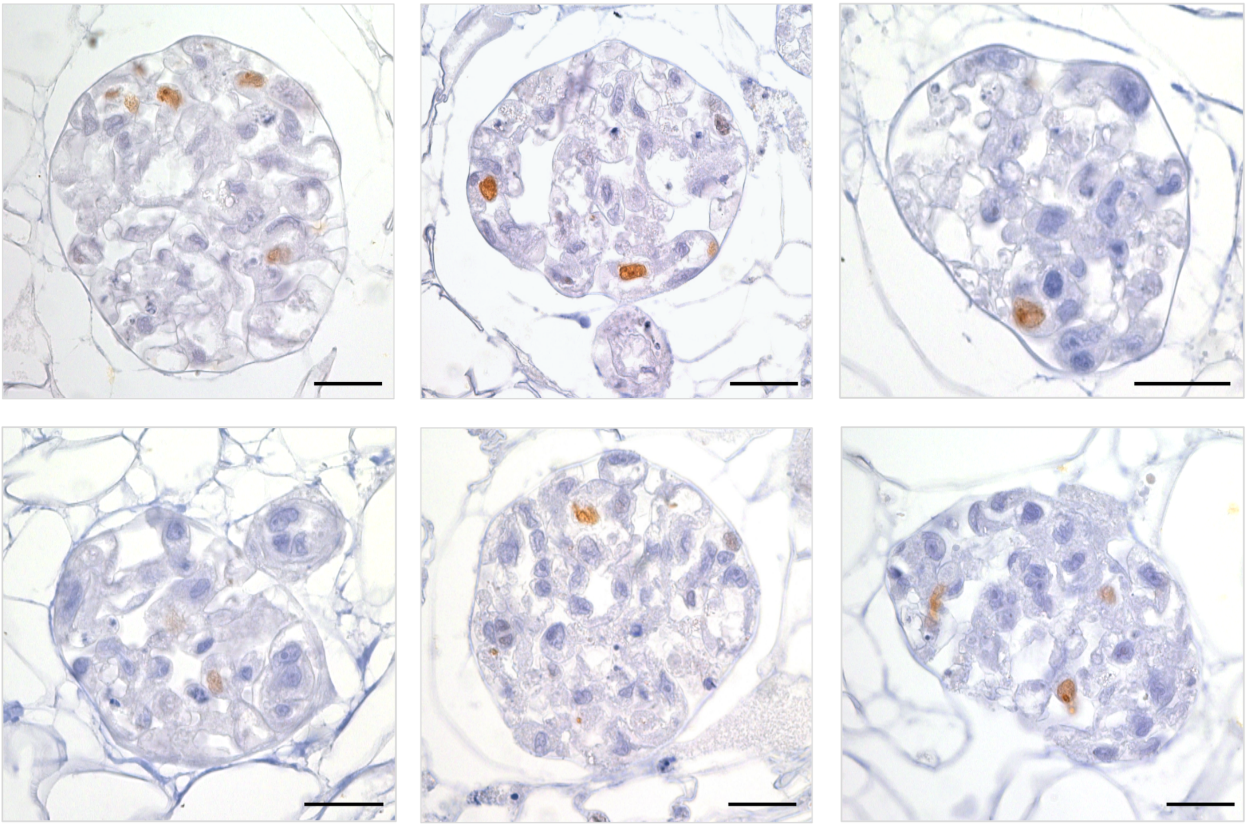

Figure S3. Ki67 immunostaining. Representative images of Ki67 staining show proliferative ability of iPSC-derived ECs after 48h infusion into rat kidney scaffold. Scale bar 20 μm.

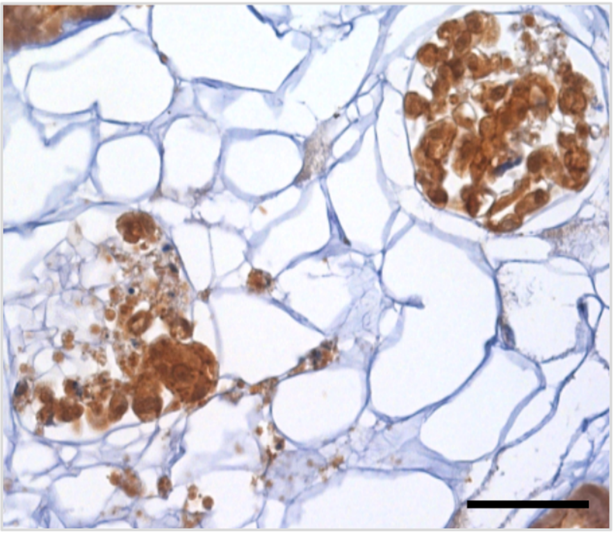

Figure S4. ZO-1 immunostaining. Representative image of ZO-1 staining shows that not all glomeruli were fully repopulated. Scale bar 50 μm.

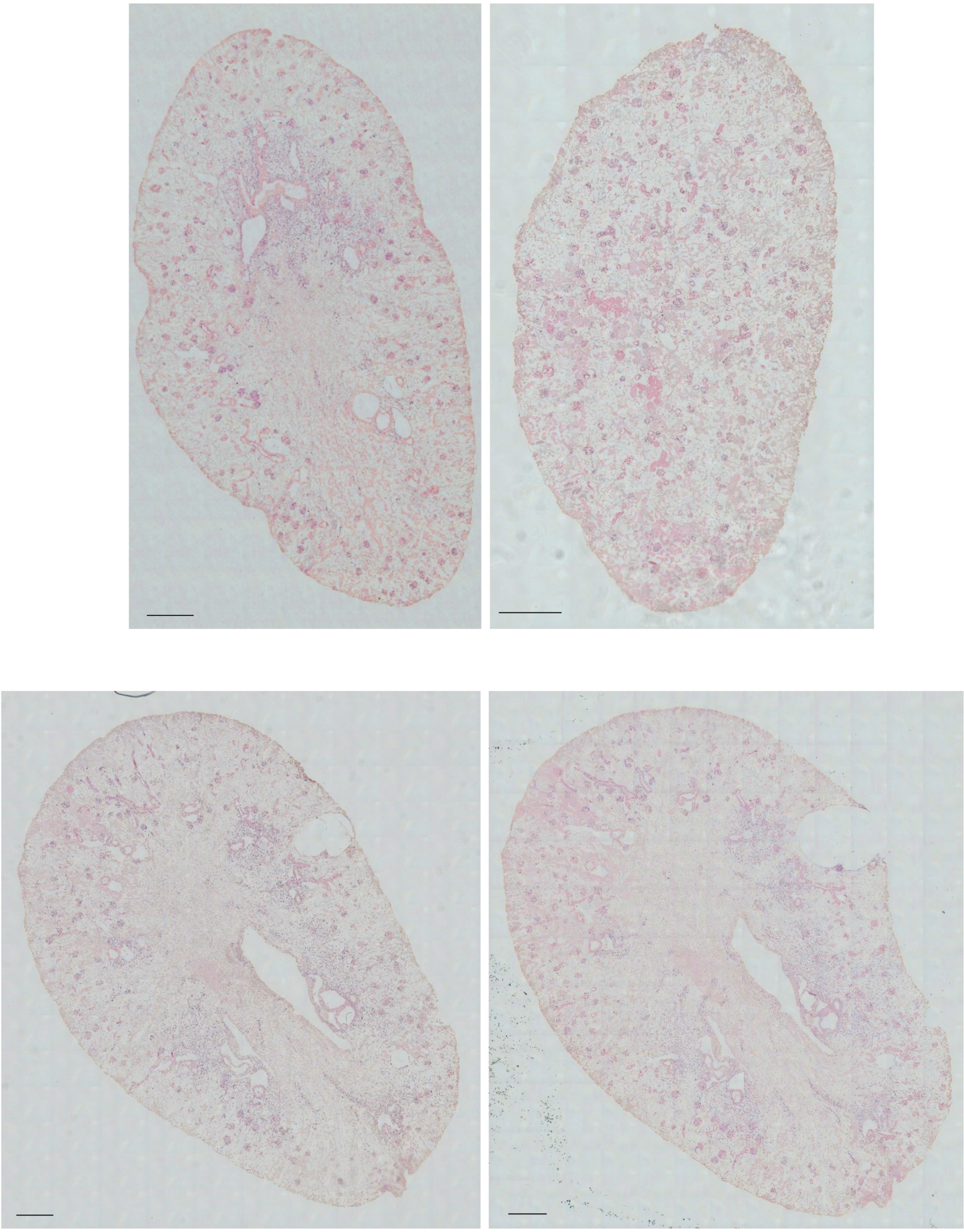

Figure S5. Reseeding of kidney scaffold with iPSC-derived ECs delivered by renal artery and vein. Mosaic view of transversal cross-sections of repopulated kidney demonstrating a homogeneous distribution of iPSC-derived ECs into glomeruli and vascular structures. Scale bar 500 μm.

Supplementary Methods

Human iPSC differentiation into endothelial cells. hiPSCs were dissociated using Accutase and plated on growth factor-reduced matrigel-(BD Biosciences) coated dishes at a density of 47.000 cells/cm^2 in mTeSR1 medium with 10 μM ROCK inhibitor Y-27632 (Sigma). After one day, medium was replaced with an induction medium consisting of a 1:1 mixture of DMEM:F12 (1:1) plus Glutamax (ThermoFisher Scientific) and Neurobasal media with N2 and B27 supplements (ThermoFisher Scientific), in the presence of 10 μM CHIR99021 (Stemgent) and 25 ng/ml BMP4 (ThermoFisher Scientific). The induction medium was maintained for three days and thereafter replaced by StemPro-34 medium (ThermoFisher Scientific) supplemented with 200 ng/ml VEGF-A (ThermoFisher Scientific) and 2 μM Forskolin (Sigma) for 2 more days in order to induce the endothelial phenotype. On day 6 of differentiation, cells were dissociated with Accutase and sorted by MACS separation to obtain pure endothelial cells. *MACS separation:* Cells on day 6 of the differentiation protocol were detached with Accutase, centrifuged and suspended into 80 μl buffer (PBS Solution pH 7.2 containing 0.5% bovine serum albumin, BSA, and 2 mM EDTA) in the presence of 20 μl CD144 antibody conjugated with micro beads (Miltenyi Biotech) for a total of 10^7 cells. After 20-minute incubation at 4°C, the cell suspension was washed by adding 2 ml of buffer and centrifuged at 300g for 10 minutes. Up to 10^8 cells were re- suspended in 500 μl of buffer and then the cell suspension was loaded onto a MACS Column, which was placed in the magnetic field of a MACS Separator. After removing the column from the magnetic field, the magnetically retained CD144$^+$ cells were eluted with 5 ml of buffer and collected as positively selected cell fraction. CD144$^+$ cells were cultured on fibronectin (2 μg/cm^2, BD Biosciences) coating dishes in StemPro-34 medium supplemented with 50 ng/ml VEGF-A (maintenance medium).

Organ decellularization. The kidney was transferred, at room temperature, into the chamber containing the solution used for organ decellularization. The cannula previously inserted into the renal artery was connected to a peristaltic pump. The kidney was perfused with a solution of 1%

sodium dodecyl sulfate (SDS; Sigma) for 6 h at a flow rate of 0.4 ml/min. Hydraulic pressure was continuously recorded by a pressure transducer (WPI, Inc), a digital data acquisition system MP150, and AcqKnowledge software (BIOPAC Systems, Inc.). Perfusion pressure was maintained within a physiological range for the entire duration of the decellularization process. Finally, the kidney was perfused with distilled water to wash out the detergent.

Table S1. List of primary antibodies

**Antigen**	**Company**	**Cat. number**	**Dilution**
OCT4	Santa Cruz Biotechnology	sc-5279	1:100
Brachyury	Abcam	ab20680	1:100
CD144	R&D System	AF938	1:100
α-SMA Cy3 conjugated	Sigma	C6198	1:100
vWF	Dako	A0082	1:400
CD31	Abcam	ab28364	1:100
Flk-1	Cell Signalling Technologies	#2479	1:100

Table S2. List of Taqman Probes

**Catalogue number**	**Gene Symbol**	**Description**
Hs99999909_m1	*HPRT1*	Homo sapiens hypoxanthine phosphoribosyl transferase 1
Hs00742896_s1	*OCT4*	Homo sapiens POU class 5 homeobox 1 (OCT4)
Hs02387400_g1	*NANOG*	Homo sapiens Nanog homeobox
Hs00610080_m1	*T(BRY)*	Homo sapiens T, brachyury homolog (mouse)
Hs00901465_m1	*CDH5(CD144)*	Home sapiens cadherin 5, type 2 (vascular endothelium)
Hs01065282_m1	*PECAM1(CD31)*	Homo sapiens platelet/endothelial cell adhesion molecule 1
